# Associations of Childhood Trauma with Paranoia and Conspiracy Thinking Among Young Adults: Exploring the Indirect Role of Attachment Styles

**DOI:** 10.3390/healthcare14060769

**Published:** 2026-03-19

**Authors:** Feten Fekih-Romdhane, Ons Ghorbel, Majda Cheour, Frederic Harb, Souheil Hallit

**Affiliations:** 1 The Tunisian Center of Early Intervention in Psychosis, Department of Psychiatry “Ibn Omrane”, Razi Hospital, Manouba 2010, Tunisia; 2 Faculty of Medicine of Tunis, Tunis El Manar University, Tunis 1068, Tunisia; 3 Faculty of Medicine and Medical Sciences, University of Balamand, Kalhat, Tripoli P.O. Box 100, Lebanon; 4 School of Medicine and Medical Sciences, Holy Spirit University of Kaslik, Jounieh P.O. Box 446, Lebanon; souheilhallit@usek.edu.lb; 5 Applied Science Research Center, Applied Science Private University, Amman P.O. Box 11931, Jordan

**Keywords:** paranoia, conspiracy beliefs, attachment styles, insecure attachment, mediation

## Abstract

**Background/Objectives**: To date, limited focus has been given to the possible contribution of attachment theory to the comprehension of how paranoia and conspiracy beliefs may develop. Our study aimed to examine the potential mediating effects of the different adult attachment styles on the relationship between childhood trauma and paranoid/conspiracy thinking. **Methods**: This is a cross-sectional study that was conducted during September–January 2025 among Tunisian young adults (aged 18–35 years) from the general population. The Child Abuse Self Report Scale (CASRS-12), the Relationship Questionnaire (RQ), the eight-item Green et al., Paranoid Thoughts Scale (GPTS-8), and the Generic Conspiracist Beliefs Scale-5 (GCB-5) were administered to participants. **Results**: After controlling for potential confounders, analyses showed that secure attachment partially mediated the link between childhood trauma and paranoia (indirect effect: Beta = 0.001; Boot SE = 0.001) and conspiracy beliefs (indirect effect: Beta = 0.024; Boot SE = 0.01). On the other hand, preoccupied attachment acted as a significant mediator in the relationship between childhood trauma and paranoid thinking (indirect effect: Beta = 0.001; Boot SE = 0.001). In all these models, greater childhood trauma was directly related to higher paranoia and/or conspiracy thinking. **Conclusions**: Findings suggest that interventions and policies aimed at promoting a more secure attachment and addressing insecure attachment representations are likely to be effective in diminishing paranoia and conspiracy beliefs, especially for victims of childhood adversity.

## 1. Introduction

A wealth of empirical research has established that paranoia exists in healthy individuals across a continuum of severity, spanning the general population at the low end and psychopathology (i.e., manifest persecutory delusions) at the high end. As such, paranoid thoughts are highly prevalent in the general population, ranging from less than 2% to nearly 30% [1]. For instance, a study found that paranoid thoughts were reported to be regularly experienced by around a third of a large general population sample recruited online [2]. Paranoia experienced by non-clinical populations refers to unfounded social evaluative fears, pervasive suspiciousness, and irrational distrust towards others whose intentions are malicious [3]. It commonly encompasses two major components, persecutory beliefs and ideas of social reference [4]. On the other hand, conspiracy theories are commonly defined as “explanations for events that implicate secretive and powerful groups who cover up information to suit their interests” ([5], p. 500). They enable people to make sense of events and cope with chaos, especially in times of crisis [6]. Due to a string of major world events and the amplification of many preexisting crises (such as the COVID-19 pandemic, armed conflicts, and climate change), conspiracy theories have sparked significant public concern and increased interest among scientists over the recent years. Indeed, because of their fast and easy propagation through online platforms, conspiracy theories gain increasing endorsement throughout the world, even if unusual, bizarre or socially unacceptable. For example, it has been documented that more than one person in four reported endorsing conspiracy beliefs in the general population of the US [7]. A systematic review encompassing 43 studies found a high but widely varying prevalence of acceptance of conspiracy theories (between 0.4 and 82.7%) in the general population during the COVID-19 pandemic [8]. The wide variation in rates of conspiracy beliefs was likely due to the presence of heterogeneity between studies in terms of methods, tools, and survey designs.

It is becoming more widely recognized and accepted that conspiracy theorizing shares some important phenomenological features with paranoia, and that some conceptual overlap exists between these two entities [9,10]. They have in common the tendency to attribute negative events or outcomes to malicious external agents, and to perceive that others make concerted efforts in their objectives to bring about negative outcomes [11]. In the conspiracism phenomenon, beliefs are shared by others and all of society is viewed as the target of persecution, whereas in paranoia beliefs are held in isolation and are rather self-referential [11]. Another conceptual similarity is that both conspiracy theories and paranoia reflect suspicious thoughts that may be difficult to falsify, and can cover theories or events that later turn out to be true [11]. Given the empirical evidence showing that the two psychopathological entities can pose significant risks to mental health, wellbeing and functioning [12,13], there is a critical need to identify their risk factors and recognize those who are at risk for or exhibit paranoid or conspiracy thinking. Conspiracy thinking and paranoia share multiple risk factors, including experiences of childhood trauma at the socio-environmental level, which will be the focus of this study.

### 1.1. The Link of Childhood Trauma to Paranoia and Conspiracy Thinking

People with experiences of traumatic events during their childhood are often subject to long-term detrimental consequences for mental health during adulthood [14,15]. In particular, a history of childhood trauma is a well-established social risk factor for both paranoia and conspiracy theories. Previous research indicated that individuals with a history of trauma are more likely to interpret major events and crises using conspiratorial frameworks, which may be largely due to three key facilitators: a sense of victimhood (engendered by the experience of violence), powerlessness (feeling deprived of personal control), and status devaluation (having less value and lower status than others) [16]. As such, when losing control, the person tends to view others as highly agentic, and when victimized, the person tends to perceive others as victimizers [16]. Growing up in adverse, potentially traumatizing childhood environments leads the individual to the adoption of hostile interpersonal styles [17], to frequently attribute hostile intent to others and to actively search for indications of conspiracy. In this regard, a recent study by Zarazińska and Jonason [18] found that stronger conspiracy believers were more likely to report an unstable, harsh, and stressful childhood. Another study demonstrated that greater exposure to childhood traumatic events was associated with increased COVID-19 vaccine-related conspiracy beliefs [19]. It has been suggested that conspiracy interpretations of the world enable trauma victims to explain the suffering they have endured [18].

On the other hand, exposure to childhood trauma was consistently found to be a strong predictor of a greater susceptibility to paranoia [20]. This aligns with the cognitive theory model of persecutory delusions, which stipulates that childhood adversity contributes to the formation of negative schema about others and the self (e.g., others seen as hostile, the self-seen as weak), which may lead, in turn, to the development of persecutory ideation [21]. Several previous studies reported a positive association between adverse events in childhood and the displaying of paranoia symptoms in adulthood [22,23]. For instance, Freeman and Fowler [24] found that the presence of a history of childhood trauma was associated with a more than two times greater likelihood of endorsing paranoid thoughts. These data raise questions about the specific processes and pathways that underline the link of trauma to paranoia and conspiracy thinking. One potential consequence of early-life traumatization that has been implicated in causing both paranoia and conspiracy mentality is insecure attachment. Hence, attachment could be a possible route by which childhood trauma history may influence paranoia and conspiracy mentality.

### 1.2. Attachment as a Mediator Between Childhood Trauma and Paranoid/Conspiracy Thinking

**Figure 1:**
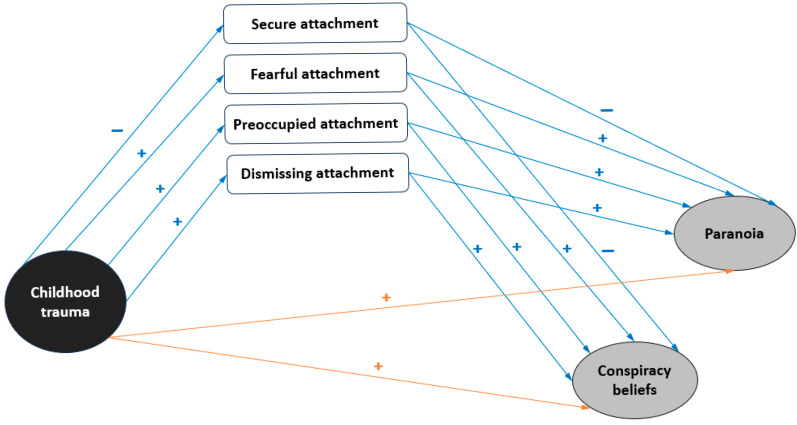
Conceptual framework of the mediation models for the present study (indirect effect = blue arrows, direct effect = orange arrows).

It is theorized that attachment representations of the self and others in relationships stem from early relationships with caregivers [25]. Experiences of responsive, sensitive and warm caregiving will lead to the formation of secure attachment patterns [26], whereas having rejecting, neglecting or insensitive caregivers contributes to the risk of developing insecure attachment patterns [27]. There exists solid evidence that attachment insecurity styles (most particularly the ‘pre-occupied’ style) play an important role in the development and persistence of paranoia symptoms, even when key confounders are controlled for [28]. At the same time, research has documented that insecure attachment is a common consequence of childhood trauma experiences [29]. Several previous research findings suggest that attachment styles develop based on childhood experiences and that childhood abuse/neglect represents significant predictors of, and developmental antecedents to, insecure attachment [30,31,32,33,34]. Grady et al. [35] have proposed an etiological framework in which childhood trauma experiences “disrupt attachment formation” (p. 434) to produce insecure attachments. Based on attachment theory positing that attachment styles develop from caregiving interactions, Grady et al. [35] claimed that childhood trauma experiences are intervening risk factors in the caregiving–attachment security association. Because some childhood traumas (such as neglect) are “attachment-disrupting”, they have the potency for developing paranoia symptoms [20]. Therefore, it can be suggested that attachment insecurity stemming from one’s personal history of childhood trauma would be correlated with conspiracy and paranoid thinking. In this context, our study proposes a conceptual framework to delve into these relationships (Figure 1).

### 1.3. Rationale and Objectives of This Study

Our study was designed to address some gaps identified in the literature. No previous studies could be found on the possible contribution of attachment theory to our understanding of how paranoia and conspiracy beliefs may develop. In addition, while the role of current living conditions in conspiracy theorizing has been the focus of considerable theoretical and empirical attention, the role of a person’s past experiences has been relatively neglected in the literature to date [18]. Furthermore, it is of note that conspiracy belief research, which seeks to understand why people hold and accept conspiracy theories, has been confined to narrow Western, high-income and peaceful societies [36]. However, there has been almost no quantitative research on the topic in non-Western parts of the world (such as Tunisia and the North-African Middle-East region), which are facing major challenges including underdevelopment, political instability, conflicts, economic crises, climate-related disasters and environmental degradation [37,38]. Therefore, our study intended to expand existing knowledge by examining the potential mediating effects of the different adult attachment styles on the relationship between childhood trauma and paranoid/conspiracy thinking.

## 2. Materials and Methods

### 2.1. Participants and Procedure

This is a cross-sectional design that was carried out during the period of September–January 2025. To be included, participants had to meet the following criteria: (1) be aged 18 to 35 years (this choice was made because the population at-risk for psychosis predominantly belongs to this age group [39]), (2) be of Tunisian nationality and residency, (3) have no previous antipsychotics intake and no personal history of a psychotic disorder diagnosed by a psychiatrist, (4) have access to the Internet, and (5) be willing to take part in the research. An online questionnaire was constructed in the Arabic language using Google Forms and distributed to collect data for this study. A combined convenience and snowball sampling technique was adopted to recruit participants. As a first step, the electronic link to the survey was circulated through social media platforms (such as Instagram, Telegram, WhatsApp, and Facebook) to the persons in contact with the investigators who are within their professional network and who met eligibility criteria (e.g., students, colleagues, staff members). Then, those who agreed to participate were encouraged to disseminate and share the link to their own personal networks. The first page of the form presented necessary information regarding the study. Before participants were asked for consent, confidentiality of personal information, the voluntary nature of participation, and the right to withdraw without penalty were clearly outlined. Ethical approval for this study was obtained from the ethics committee of Razi Psychiatric Hospital, Manouba, Tunisia.

### 2.2. Minimal Sample Size Calculation

According to Fritz and MacKinnon [40], a minimal sample of 412 was deemed necessary based on this formula: $n=\frac{L}{f2}+k+1$, where f = 0.14 for small effect size, L = 7.85 for an α error of 5% and power β = 80%, and k = 10 variables to be entered in the model.

### 2.3. Assessment Instruments

#### 2.3.1. Demographics

Participants were asked to self-report their sex (Male, Female), age, marital status (married, single, divorced, widowed), educational attainment (primary, secondary, university), living arrangement (with family, with friends, alone), tobacco use (yes, no), alcohol consumption (yes, no), lifetime cannabis use (yes, no), and personal history of diagnosed mental illness other than psychotic disorders (yes, no). In addition, the household overcrowding index was calculated (the ratio of the total number of people over the total number of rooms in the house except the bathrooms and kitchen) as an estimate of overall socioeconomic status. Higher ratios designate lower socioeconomic status [41].

#### 2.3.2. The Child Abuse Self Report Scale (CASRS-12)

The CASRS-12 is validated in the Arabic language [42], and represents a shortened version of the original 38-item CASRS [43]. It measures four categories of childhood trauma: psychological (3 items, e.g., “My parents blame me in others’ presence”), neglect (3 items reverse scored, e.g., “My family pays attention to my wishes”), physical abuse (3 items, e.g., “If I do not obey the rules of my family, I will be punished very hard”), and sexual abuse (3 items, e.g., “An adult or some adults have tried to touch my private parts”). Items are rated on a four-point scale ranging from 0 (Never) to 3 (Always). Higher total scores reflect more severe childhood trauma experiences. In the present study, the Cronbach’s α value was 0.83.

#### 2.3.3. The Relationship Questionnaire (RQ)

The RQ is composed of four items used to assess four different attachment styles: secure, fearful, preoccupied, and dismissing [44]. Each item is designed in the form of a small paragraph that describes a prototypical attachment pattern applying to adult close relationships. For example, secure attachment is assessed through the following item: “It is easy for me to become emotionally close to others. I am comfortable depending on them and having them depend on me. I don’t worry about being alone or having others not accept me” and preoccupied attachment is measured through the following item: “I am uncomfortable getting close to others. I want emotionally close relationships, but I find it difficult to trust others completely, or to depend on them. I worry that I will be hurt if I allow myself to become too close to others”. Respondents are asked to read and rate their agreement with each statement on a 7-point scale ranging from 1 (Not at all like me) to 7 (Very much like me). Higher scores reflect greater endorsement of each of the attachment orientations. The Arabic validated version of the RQ [45] was used in this study.

#### 2.3.4. The Eight-Item Green et al., Paranoid Thoughts Scale (GPTS-8)

The GPTS-8 was developed by Bianchi and Verkuilen [46] as a short version of the 18-item Revised GPTS (R-GPTS) [47]. The scale is intended to assess paranoia symptoms over the past 30 days. The scale contains eight items that are divided into two 4-item dimensions: “Ideas of social reference” (e.g., “I spent time thinking about friends gossiping about me”) and “Ideas of persecution” (e.g., “People have been hostile toward me on purpose”). Items can be rated on a five-point scale from 1 (“Not at all”) to 5 (“Totally”). Higher scores reflect higher paranoid thinking [46]. The Arabic validated version of the GPTS-8 was used in this study [48], which yielded a Cronbach α of 0.83 for each of the two factors.

#### 2.3.5. The Generic Conspiracist Beliefs Scale-5 (GCB-5)

The GCB-5 [49] is a shortened form of the Generic Conspiracist Beliefs Scale (GCB-15; [50]). The scale was shown to be a valid and reliable measure of conspiracy beliefs. It is composed of five items (e.g., “Experiments involving new drugs or technologies are routinely carried out on the public without their knowledge or consent”). Response options for each item vary from 1 (Definitely not true) to 5 (Definitely true). Greater scores indicate greater beliefs in, and acceptance of conspiracy theories [49]. The Arabic validated version of the GCB-5 was used in this study, which yielded a Cronbach α of 0.75 for total scores.

### 2.4. Statistical Analysis

The SPSS software v.25 was used for the statistical analysis. The paranoia score was not normally distributed but the LOG transformation fulfilled the before-mentioned conditions, which was used throughout the analysis. The normality of the distribution of the generic conspiracist beliefs score was confirmed by calculating the skewness and kurtosis; skewness and kurtosis values between −1 and +1 are considered acceptable to prove a normal univariate distribution. The Student *t* test was used to compare two means, and Pearson’s test to correlate two continuous variables. The PROCESS Macro version 4.2, model 4 software was used to conduct the mediation analysis, taking childhood trauma as the independent variable, adult attachment, interpersonal trust and threat sensitivity as the mediators and paranoid thoughts and generic conspiracist beliefs as the dependent variables. We tested separate mediation models because each attachment style was examined as an independent mediator (single-mediator model 4). This approach was chosen to estimate the indirect effect for each attachment style separately and to avoid overlap/suppression effects that can arise when entering multiple correlated attachment dimensions simultaneously in the same regression-based mediation model. The indirect effect was deemed significant if the confidence interval did not pass through zero. Results were adjusted for all variables that showed a *p* < 0.25 in the bivariate analysis. *p* < 0.05 was deemed statistically significant.

## 3. Results

### 3.1. Sample Characteristics

Five hundred fifteen young adults took part in this study, with a mean age of 25.45 ± 4.04 years. The vast majority were unmarried (85.4%) and had a university level of education (98.0%). Other descriptive statistics of our sample are presented in Table 1.

### 3.2. Bivariate Analysis of Factors Associated with Paranoia Thoughts and Conspiracist Beliefs

The results showed that tobacco use was significantly associated with higher paranoia thoughts. Being a lifetime cannabis user was significantly associated with higher conspiracist beliefs, more childhood trauma experiences, greater endorsement of fearful and preoccupied attachment styles, and lower endorsement of secure attachment (Table 2). Moreover, higher conspiracist beliefs were significantly associated with higher childhood trauma and lower secure attachment (Table 3).

### 3.3. Mediation Analysis Taking Paranoia Thoughts as the Dependent Variable

**Figure 2:**
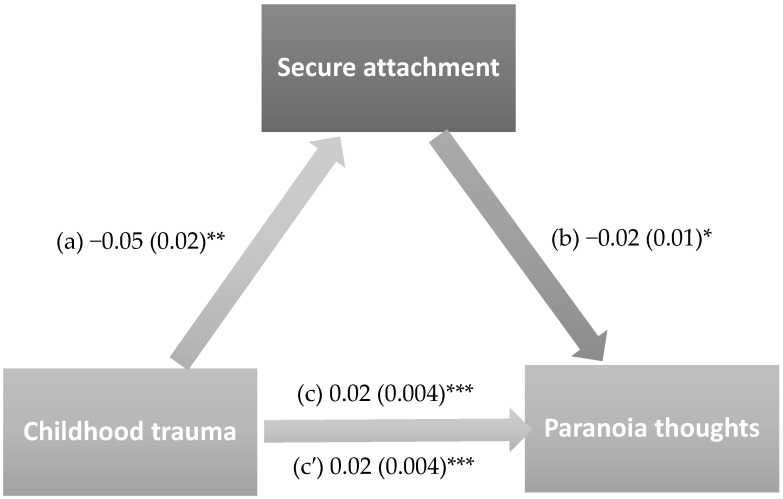
The mediation model with secure attachment as a mediator. (a) Relation between childhood trauma and secure attachment (R^2^ = 0.036); (b) Relation between secure attachment and paranoia thoughts (R^2^ = 0.080); (c) Total effect of childhood trauma and paranoia thoughts (R^2^ = 0.070); (c′) Direct effect between childhood trauma and paranoia thoughts. Numbers refer to unstandardized Beta values and their standard errors. * *p* < 0.05; ** *p* < 0.01; *** *p* < 0.001.

**Figure 3:**
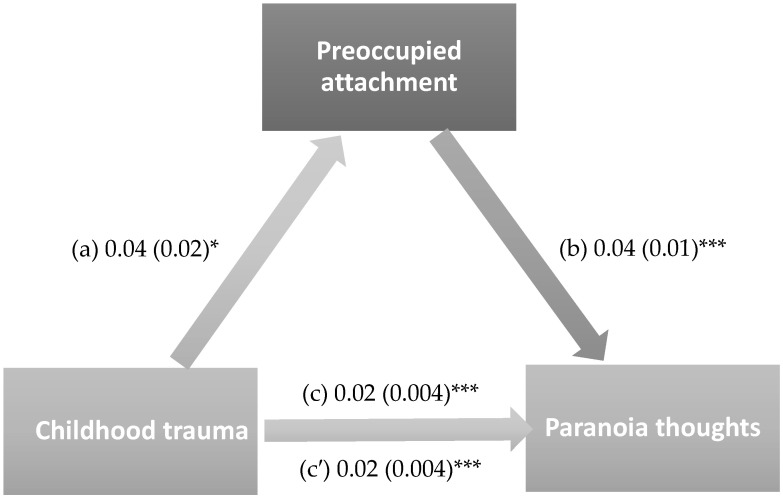
The mediation model with preoccupied attachment as a mediator. (a) Relation between childhood trauma and preoccupied attachment (R^2^ = 0.047); (b) Relation between preoccupied attachment and paranoia thoughts (R^2^ = 0.104); (c) Total effect of childhood trauma and paranoia thoughts (R^2^ = 0.070); (c′) Direct effect between childhood trauma and paranoia thoughts. Numbers refer to unstandardized Beta values and their standard errors. * *p* < 0.05; *** *p* < 0.001.

The results of the mediation analysis were adjusted over the following variables: sex, marital status, household crowding index, tobacco use, and personal history of mental illness. Secure (indirect effect: Beta = 0.001; Boot SE = 0.001; Boot 95% CI 0.00, 0.003) and preoccupied attachment (indirect effect: Beta = 0.001; Boot SE = 0.001; Boot 95% CI 0.00, 0.003) partially mediated the association between childhood trauma and paranoia thoughts. Higher childhood trauma was significantly associated with lower secure attachment and higher preoccupied attachment, whereas higher secure attachment was significantly associated with lower paranoia thoughts and higher preoccupied attachment was significantly associated with higher paranoia thoughts. Finally, higher childhood trauma was directly associated with higher paranoia thoughts (Figure 2 and Figure 3). Moreover, 6.4% and 8.4% of the total effect were mediated through secure and preoccupied attachment respectively.

Fearful (indirect effect: Beta = 0.00; Boot SE = 0.001; Boot 95% CI −0.002, 0.002) and dismissing attachments (indirect effect: Beta = 0.0001; Boot SE = 0.0003; Boot 95% CI −0.004, 0.0009) did not mediate the association between childhood trauma and paranoia thoughts.

### 3.4. Mediation Analysis Taking Conspiracist Beliefs as the Dependent Variable

**Figure 4:**
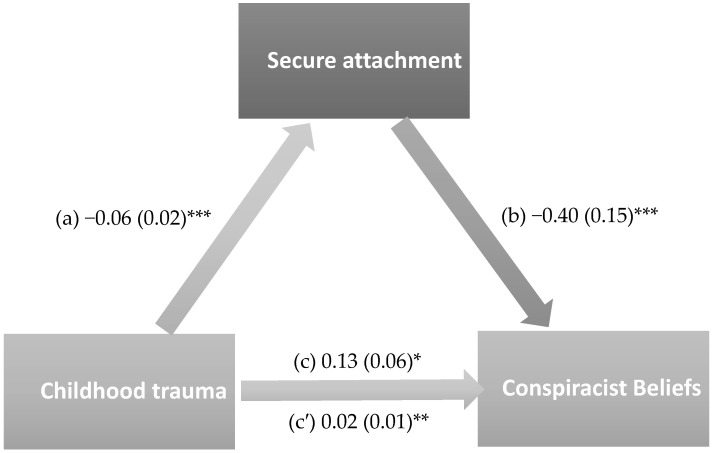
The mediation model with secure attachment as a mediator. (a) Relation between childhood trauma and secure attachment (R^2^ = 0.039); (b) Relation between secure attachment and generic conspiracist beliefs (R^2^ = 0.057); (c) Total effect of childhood trauma and generic conspiracist beliefs (R^2^ = 0.044); (c′) Direct effect between childhood trauma and generic conspiracist beliefs. Numbers refer to unstandardized Beta values and their standard errors. * *p* < 0.05; ** *p* < 0.01; *** *p* < 0.001.

The results of the mediation analysis were adjusted over the following variables: sex, age, educational attainment, household crowding index, tobacco use, lifetime cannabis use, and personal history of mental illness. Secure attachment (indirect effect: Beta = 0.024; Boot SE = 0.01; Boot 95% CI 0.01, 0.05) partially mediated the association between childhood trauma and conspiracy beliefs. Higher childhood trauma was significantly associated with lower secure attachment, whereas higher secure attachment was significantly associated with lower generic conspiracist beliefs. Finally, higher childhood trauma was directly associated with higher conspiracist beliefs (Figure 4). Moreover, 18.8% of the total effect was mediated through secure attachment.

Fearful (indirect effect: Beta = 0.004; Boot SE = 0.005; Boot 95% CI −0.003, 0.016), preoccupied (indirect effect: Beta = −0.0001; Boot SE = 0.01; Boot 95% CI −0.01, 0.01) and dismissing attachment styles (indirect effect: Beta = −0.003; Boot SE = 0.005; Boot 95% CI −0.01, 0.004) did not mediate the association between childhood trauma and conspiracist beliefs.

## 4. Discussion

Paranoid and conspiracist belief systems are influenced by multiple socio-environmental determinants, such as exposure to traumas and adverse events occurring during childhood. To contribute to previous research efforts, the purpose of this research study was to examine the mediating effect of attachment styles on conspiracy beliefs and paranoia. Our findings replicate those of previous research by showing that childhood trauma had a significant and positive direct effect on both paranoia and conspiracy beliefs. In addition, the indirect effects through insecure attachment styles were significant.

Mediation models showed that direct effects were significant, suggesting that individuals who experienced childhood trauma were more inclined to adopt a conspiratorial mindset and endorse paranoid thinking. This aligns with the existing evidence that early-life trauma experiences may represent a psychological precursor of the holding of conspiracy beliefs [18,19] and the presence of paranoia [22,23,24]. Therefore, our findings provide further support to the idea that conspiracy and paranoia thinking can be a response to living (or surviving) in a complex, unsafe and threatening world [18]. In this regard, a Polish study found that endorsement of popular conspiracy beliefs was associated with more difficult childhood conditions, suggesting that belief systems can be regarded as “sensible if not logical responses” activated by early-life challenging experiences [18].

In terms of indirect effects, our results demonstrated that both secure and preoccupied attachment orientations partially mediated the associations between childhood trauma and paranoia, whereas only secure attachment acted as a significant mediator between childhood trauma and conspiracy beliefs. In other words, people who were victimized during infancy tended to have more trouble developing secure relationships with others, which was, in turn, associated with more endorsement of paranoia and conspiracy beliefs. These findings reflect the core nature of attachment; that is, people who developed a ‘secure’ attachment style tend to build and value close, long-lasting relationships [51]. In contrast, those who developed a ‘preoccupied’ attachment style tend to experience intense worry about their relationships and fear of abandonment or separation. Interestingly, the present results concur with previous observations that the role of trauma in paranoia is non-specific and rather indirect, whereby exposure to trauma is likely to create a range of psychological processes, including negative ideas about the self and others, which are established causal factors for the development of paranoia [23]. Moreover, and broadly in line with our results, a study among adults with self-reported psychotic disorders from the United Kingdom revealed that disorganized attachment mediated the association between childhood interpersonal trauma and paranoia [52].

Preoccupied attachment played a role in the link of childhood trauma to paranoia thoughts but not to conspiracist beliefs. This could be explained by the fact that conspiracy ideation is collective, ideological (sociopolitical), and system-oriented, whereas paranoia is interpersonal and self-focused. In attachment theory, individuals with a preoccupied attachment style tend to ruminate about relationship-related concerns and amplify interpersonal threat cues [53]. Therefore, while this attachment style can contribute to distrust in close relationships, it may not necessarily affect generalized ideological thinking. Altogether, childhood trauma is assumed to be an environmental stressor which is associated with a higher tendency to engage in paranoid and conspiracy beliefs, especially in people with insecure attachment styles.

Across the mediation models that considered paranoia as the dependent variable, secure and preoccupied attachment showed small but significant indirect effects, accounting for approximately 6% and 8% of the total association, respectively. These findings indicate that attachment contributes modestly to the trauma–paranoia relationship, with most of the association remaining direct or explained by other mechanisms. In contrast, secure attachment played a more substantial role in the model predicting conspiracy beliefs, accounting for nearly 19% of the total effect. Although the unstandardized indirect effects appear numerically small, partly due to the LOG transformation of the paranoia score, proportion-mediated numbers clarify that attachment represents a modest but theoretically meaningful mechanism, particularly for conspiracy beliefs. These results underscore the importance of distinguishing statistical from substantive significance.

### 4.1. Study Limitations

Our study has a few notable limitations that need to be considered and addressed in future research. First, the study has a cross-sectional design, which does not allow us to draw causal inferences between childhood trauma and attachment. Second, the study relied on online self-administered questionnaires, which might question the reliability of findings, especially with regard to the reporting of paranoia symptoms [54]. Third, retrospective self-reporting of childhood trauma may allow room for potential recall bias. Fourth, the snowball sampling method used to gather participants can limit the generalizability of results, as it may have excluded older participants and those with lower levels of education. Fifth, because there might be a conceptual overlap between paranoia and conspiracy beliefs [9,10], future studies considering the application of a multivariate or latent variable approach could help refine the theoretical understanding of the relationships between study variables and estimate effects more reliably. Finally, the RQ was used to measure attachment styles. Due to its single-item format, it can be of limited reliability and validity and may not fully reflect the complexity of the attachment construct. Future studies employing a multi-item measure of attachment styles could provide more robust measurement and more complete information about the construct.

### 4.2. Clinical and Research Implications

We believe our study adds new knowledge and a better understanding of the direct and indirect roles that personal childhood trauma experiences might play in shaping how one approaches others and the world. Findings shed light on the significance of considering both the exposure to childhood trauma and attachment styles in the development of paranoia and conspiracy thinking. Therefore, routine assessments of trauma history and attachment styles could benefit help-seekers who hold beliefs in paranoid or conspiratorial ideas. Our mediation findings suggest that the role of attachment styles seems to have relevant clinical implications for the prevention and treatment of paranoid and conspiracy thinking. Interventions and policies aimed at promoting a more secure attachment and addressing preoccupied attachment representations are likely to be effective in reducing paranoia and conspiracy beliefs, especially for individuals with a history of childhood trauma [55]. Although attachment representations formed early in life remain relatively stable and influential in adult life [56], they also are shown to be malleable with interpersonal experiences and as a result of psychotherapy. Indeed, psychological therapy can offer the individual an opportunity to change their negative views of the self and/or others and experience new interpersonal relationships that deviate from early attachment representations, thus promoting the attainment of secure attachment [57,58]. Such strategies focused on encouraging more positive views of the self and others and reducing negative ideas showed promise in decreasing paranoid and suspicious thoughts [59]. As for research perspectives, our findings provide justification for further longitudinal studies to confirm any causal directions. In addition, interventional research is needed to clarify how interventions targeting a history of trauma and insecure attachment styles could influence the formation and maintenance of paranoia and conspiracy thinking in adulthood. Finally, as trauma may influence paranoid and conspiracy thinking in different ways, studies exploring the effects of other moderators/mediators are encouraged. Such studies may help uncover the pathogenesis of these psychopathological phenomena in a more comprehensive view, and offer new targets for treatment.

## 5. Conclusions

This study aimed to test a theory-driven model attempting to explain the mechanisms involved in the relationship between childhood trauma and paranoia/conspiracy beliefs. The mediation analyses showed that paranoia and conspiracy beliefs are both directly and indirectly related to childhood trauma, suggesting an indirect effect through insecure attachment. As such, results support the incorporation of attachment-based therapies into intervention plans for individuals with a history of trauma who present with current paranoia symptoms or conspiracy beliefs. Future studies are required to test the current model using longitudinal data and confirm causality.

## Figures and Tables

**Table 1:** Sociodemographic and other characteristics of the sample (*n* = 515).

Variable	*n* (%)
Sex	
Male	160 (29.6%)
Female	381 (70.4%)
Marital status	
Married	79 (14.6%)
Unmarried (Single/Divorced/Widowed)	462 (85.4%)
Educational attainment	
Primary/Secondary	11 (2.0%)
University	530 (98.0%)
Living arrangement	
With family	387 (71.5%)
With friends	71 (13.1%)
Alone	83 (15.3%)
Tobacco use	
No	450 (83.2%)
Yes	91 (16.8%)
Alcohol consumption	
No	435 (80.4%)
Yes	106 (19.6%)
Lifetime cannabis use	
No	456 (84.3%)
Yes	85 (15.7%)
Personal history of diagnosed mental illness (other than psychotic disorders)	
No	492 (90.9%)
Yes	49 (9.1%)
Age (years)	25.45 ± 4.04 [min = 18; max = 35]
Household crowding index (person/room)	1.14 ± 0.52
Paranoia thoughts (GPTS-8 scores)	6.51 ± 6.51
Conspiracy beliefs (GCB-5 scores)	16.42 ± 6.08
Childhood trauma	5.53 ± 5.01
Secure attachment	3.66 ± 1.81
Fearful attachment	3.35 ± 1.89
Preoccupied attachment	2.64 ± 1.77
Dismissing attachment	3.80 ± 2.03

GPTS-8: Green et al., Paranoid Thoughts Scale-8; GCB-5: Generic Conspiracist Beliefs scale-5.

**Table 2:** Bivariate analyses of factors associated with paranoia thoughts and conspiracist beliefs scores.

	Paranoia Thoughts			Conspiracist Beliefs		
Variable	Mean ± SD	*p*	Effect Size	Mean ± SD	*p*	Effect Size
Sex		0.221	0.125		0.191	0.123
Male	0.76 ± 0.39			16.88 ± 6.02		
Female	0.71 ± 0.39			16.13 ± 6.10		
Marital status		0.081	0.228		0.528	0.077
Married	0.65 ± 0.36			16.75 ± 6.18		
Single	0.74 ± 0.40			16.28 ± 6.07		
Educational attainment		0.790	0.059		0.090	0.517
Primary/Secondary	0.75 ± 0.23			13.27 ± 5.90		
University	0.72 ± 0.39			16.41 ± 6.07		
Tobacco use		**0.012**	0.314		0.224	0.140
No	0.70 ± 0.39			16.20 ± 6.03		
Yes	0.83 ± 0.41			17.05 ± 6.34		
Alcohol consumption		0.898	0.015		0.335	0.104
No	0.72 ± 0.39			16.22 ± 5.99		
Yes	0.73 ± 0.40			16.86 ± 6.43		
Lifetime cannabis use		0.556	0.075		**0.013**	0.294
No	0.72 ± 0.38			16.07 ± 5.99		
Yes	0.75 ± 0.44			17.85 ± 6.38		
Personal history of mental illness		0.081	0.277		0.058	0.285
No	0.71 ± 0.39			16.19 ± 6.07		
Yes	0.82 ± 0.41			17.92 ± 6.08		

Numbers in bold indicate significant *p* values.

**Table 3:** Pearson correlation matrix.

	1	2	3	4	5	6	7	8
1. Paranoid thoughts	1							
2. Conspiracist Beliefs	0.11 *	1						
3. Age	−0.04	−0.06	1					
4. Household overcrowding index	0.03	0.08	−0.09 *	1				
5. Childhood trauma	0.28 ***	0.13 **	0.09 *	0.07	1			
6. Secure attachment	−0.15 ***	−0.11 **	−0.07	−0.01	−0.13 **	1		
7. Fearful attachment	0.27 ***	0.08	−0.17 ***	0.02	0.05	−0.20 ***	1	
8. Preoccupied attachment	0.27 ***	0.04	−0.13 **	0.13 **	0.13 **	−0.04	0.42 ***	1
9. Dismissing attachment	−0.03	0.05	−0.06	0.01	−0.05	−0.09 *	0.27 ***	0.12 **

* *p* < 0.05; ** *p* < 0.01; *** *p* < 0.001.

## Data Availability

The datasets generated and/or analyzed during the current study are not publicly available due to restrictions from the ethics committee but are available from the corresponding authors on reasonable request.
